# Clinical Features for Mild Hand, Foot and Mouth Disease in China

**DOI:** 10.1371/journal.pone.0135503

**Published:** 2015-08-24

**Authors:** Baoyan Liu, Lin Luo, Shiyan Yan, Tiancai Wen, Wenjing Bai, Hongjiao Li, Guoliang Zhang, Xiaoying Lu, Yan Liu, Liyun He

**Affiliations:** 1 China Academy of Chinese Medical Sciences, Beijing, China; 2 Institute of Basic Research in Clinical Medicine, China Academy of Chinese Medical Sciences, Beijing, China; 3 Department of Infectious Diseases, First Affiliated Hospital of Anhui University of Traditional Chinese Medicine, Hefei, China; 4 Traditional Chinese Medicine Data Centre, China Academy of Chinese Medical Sciences, Beijing, China; University of Alabama at Birmingham, UNITED STATES

## Abstract

**Background:**

Mild hand, foot and mouth disease (HFMD) is at a critical stage owing to its ease of communicability and a higher risk of developing severe complications and death. Clinical diagnosis of mild HFMD was made by the presenting symptoms and signs (symptoms in brief) alone. We aim to evaluate the frequencies of symptoms in a retrospective case series study.

**Methods:**

We collected epidemiological, demographic, clinical, and laboratory data from outpatient and inpatient settings on the clinical data warehouse system. We principally described the frequencies of symptoms of mild HFMD. Correlations between symptoms with laboratory-confirmed cases were then analyzed.

**Results:**

The clinical data warehouse system included 3649 probable cases, between 2010 and 2012, of which 956 (26.20%) were laboratory confirmed. The peak incidence was identified in children 2 years of age. A total of 370 of the 956 laboratory confirmed cases (38.70%) were associated with enterovirus 71 (EV71). Logistic regression analysis adjusted for geographical variables, age, sex, month of onset, and time from onset to diagnosis showed that the clinical features constipation (*P*<0.0001; adjusted OR, 95%CI (2.99, 2.28–3.91)), and blisters (*P*<0.0001; adjusted OR, 95%CI (2.16, 1.82–2.56)) were positively correlated with the confirmed cases.

**Conclusions:**

This is the largest case series study, including all the guideline-mentioned symptoms of mild HFMD. Our findings suggest that blisters and constipation should be considered as potential warning signs while front-line clinicians manage surges of children diagnosed with mild HFMD during a pandemic.

## Introduction

Epidemics of hand, foot and mouth disease (HFMD) have been documented in North America, Australia, and Europe since it was first reported in Toronto, Canada in 1957 [1,2,3]. Historically, outbreaks of HFMD have been scattered and regional, but this pattern has changed with 2628 cases reported in Malaysia in 1997, nearly 130,000 cases in Taiwan in 1998, and several thousand cases in Singapore in 2000 [4,5,6]. Since then, HFMD has been continuously observed in the Asia-Pacific region [6]. In 2008, the disease was listed as a class “C” notifiable disease in China. In recent years, the number of HFMD cases has been increasing rapidly in Mainland China [7,8]. The cumulative total of reported cases in China reached 1.7 million in 2010, nearly 1.9 million in 2013, and 2.7 million cases in 2014 marking an era of unprecedented large-scale outbreaks in the Asia-Pacific area [6,9]. However, effective chemoprophylaxis or vaccination approaches for dealing with HFMD are still not available [10,11]. Clinical management of HFMD is largely supportive in nature.

Symptoms play significant role for clinical diagnosis and treatment. Zhou *et al*. used a large-scale biomedical literature database to construct a symptom-based human disease network and investigated the connection between symptoms and their underlying molecular interactions [12]. In addition, Li et al. explored the correlations between symptoms and herbs in large-scale real-world clinical data, which would be helpful for treatment [13]. Early recognition of warning signs of the illness as well as evaluation of probable HFMD are based on symptoms alone and are important in identifying patients that are likely to develop a severe form of the disease [14]. In some early series, recognition of warning signs focused mainly on complications of HFMD [15,16]. In other series, symptoms were reported in patients with specific viruses of HFMD, such as enterovirus 71 (EV71), Coxsackie virus A16 (CV-A16), and others [17,18]. In China, patients with HFMD, whether probable or confirmed, were classified as severe if they had any neurological complications or cardiopulmonary complications, or both; otherwise, patients were categorized as mild cases. Thus, mild HFMD is at the critical stage for preventing further deterioration. Clinical diagnosis of mild HFMD was made by the presenting symptoms alone [14]. However, few studies have examined the symptoms of mild HFMD according to the guidelines.

We analyzed the symptoms on clinical data warehouse system (CDWs) of patients with mild HFMD. This study aims to identify whether overlooked symptoms existed in the guidelines issued by China (2010ed.).

## Methods

### Clinical data warehouse system

The clinical data warehouse system consisted of the State Administration of Traditional Chinese Medicine of China (SATCM) approved special research projects for the Chinese Medicine industry including the prevention and treatment of HFMD, and required to develop the CDWs [19]. In 2010, The CDWs was designed based on Java and J2EE platforms established by the China Academy of Chinese Medical Sciences (CACMS) for supporting the medical knowledge discovery and clinical decision-making. The CDWs incorporates the structured electronic medical record (SEMR) as the core data source, an extraction-transformation-loading (ETL) tool implemented to integrate and normalize the clinical data from different operational data sources [20]. The CDWs included the cases from 33 infectious departments in 21 provinces, in departments that are located in both secondary and tertiary hospitals.

### Data definitions and collection

A probable case of HFMD was defined as a patient fulfilling the clinical features for mild HFMD (without complications, China 2010ed.) [14]. Clinical features of mild HFMD were guided by fever, skin papular or vesicular rash on hand, foot, mouth or buttock, blister (the clear liquid or pus inside the vesicular rash), which was at times accompanied by cough, rhinorrhea and loss of appetite. A few patients may not develop fever. The above-mentioned symptoms were labeled *Guide Group*. A confirmed case was defined as a clinically diagnosed case with laboratory evidence of enterovirus infection (including EV71 and/or CV-A16, or other) detected by RT-PCR, real-time PCR, or virus isolation [21].

Upon diagnosis of mild HFMD, our study included the following individual information: age (0–14 years), gender, birth date, hospital name, symptom onset date, diagnosis date, and skin rash regions. In the CDWs design process, the symptoms were determined based on typical cases and discussion by clinical experts [22]. Our previous study showed that these symptoms in addition to the guide group were important, including sialorrhea, constipation, lethargy, dysphoria, vomiting, sputum and diarrhea. The above-mentioned symptoms were labeled *Potential Warnings Group*.

### Data description

Based on the CDWs, we principally described the symptoms of mild HFMD in China. First, we characterized the distribution of cases as a function of space and age. We analyzed demographic and clinical features of patients with mild HFMD, combining probable and confirmed cases.

Second, we established an arc diagram that graphical displays the networks of symptoms in a one-dimensional layout. The main idea is to display symptoms (nodes) along a single axis, while representing the edges or connections between nodes with arcs. Each feature is connected by an arc if they appear together in the same patient, and the wider the arc, the more the features appeared in patients together. The color (and size) of the nodes identifies groups (and frequency) of features that affected patients together [23].

Third, we analyzed the frequencies of symptoms of mild HFMD in probable and confirmed cases. Then, we used logistic regression to estimate the association of symptoms with confirmed cases, after adjusting for geographical variables, age, sex, month of onset, and time from onset to diagnosis. ArcGIS v9.0 (ESRI, Redlands, CA, USA) was used to visualize the spatial distribution of mild HFMD cases. All statistical procedures were performed with R package (R Development Core Team, 2013,http://www.R-project.org/).

### Ethics statement

This CDWs project is authorized by the SATCM. Institute of Basic Research in Clinical Medicine, China Academy of Chinese Medical Science (IBRCM-CACMS) having been allowed to access to the clinical data warehouse system. To keep the data anonymous during analysis, before analysis, special people removed some field of interest that was originally collected. Here, they omitted patient name, location, phone numbers, ID number and date of birth that has been collected. We sought ethical approval for this study. The data source and study was approved by the Ethics Committee of IBRCM-CACMS (2014 No. 24). The Ethics Committee waived the need for individual informed consent due to the retrospective nature of the study. Persons are only approved access to data after administrative approval of a formal analysis request, and it will be inspected to make sure that all output do not involve the sensitive information. All patient records/information was anonymized and de-identified prior to analysis.

## Results

### Geographic distribution

**Fig 1:**
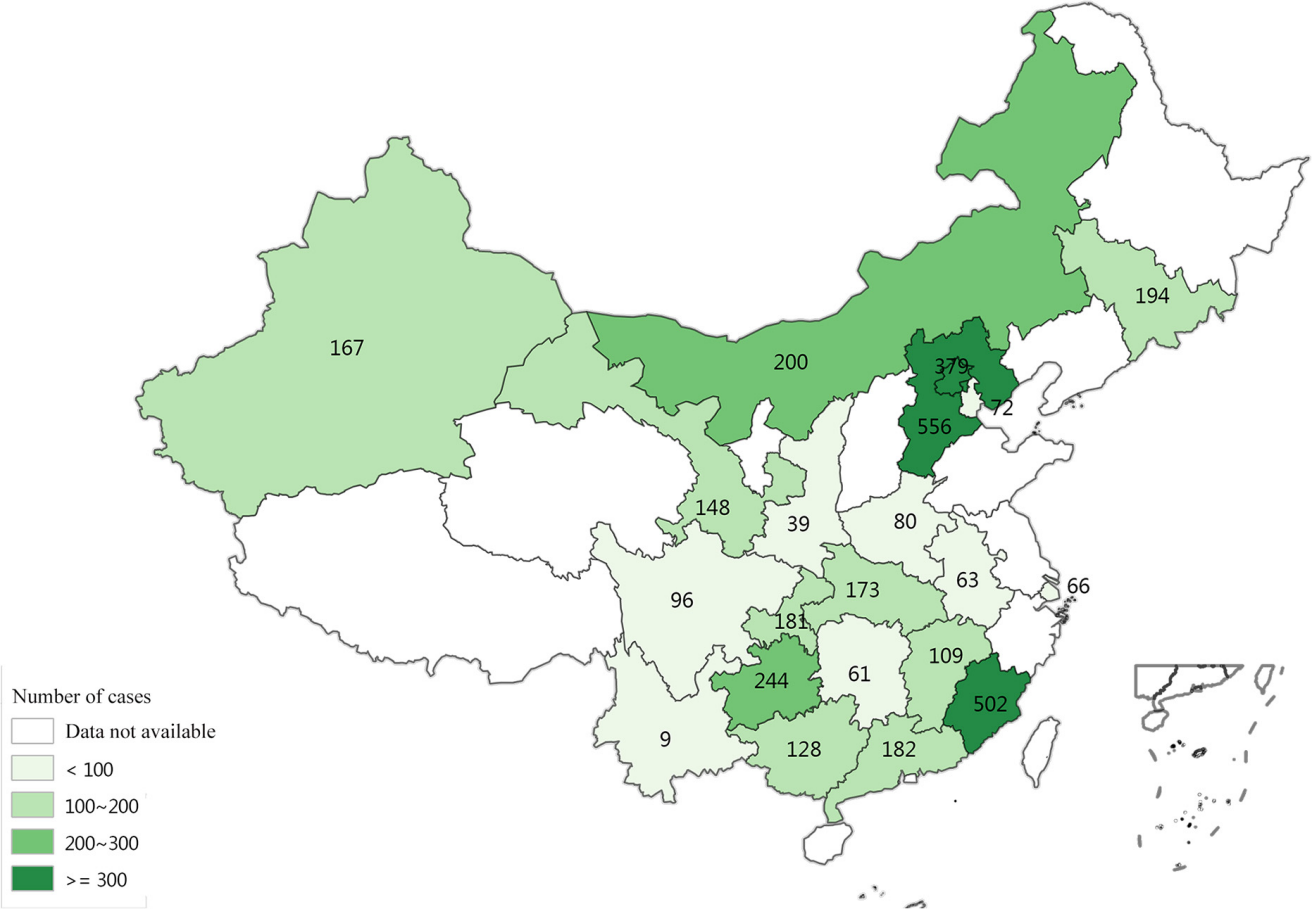
Geographical distribution of probable cases and laboratory-confirmed cases of mild HFMD by Chinese province.

A total of 3649 cases, who were clinically diagnosed with mild HFMD during 2010–2012, had been extracted from the CDWs. Fig 1 shows the geographic distribution of mild HFMD cases. The majority of cases were recorded in the main provinces of China such as Hebei and Fujian (S1 Table). The data in Fig 1 did not mean that they presented the total number of HFMD cases in the corresponding region.

### Population distribution

**Fig 2:**
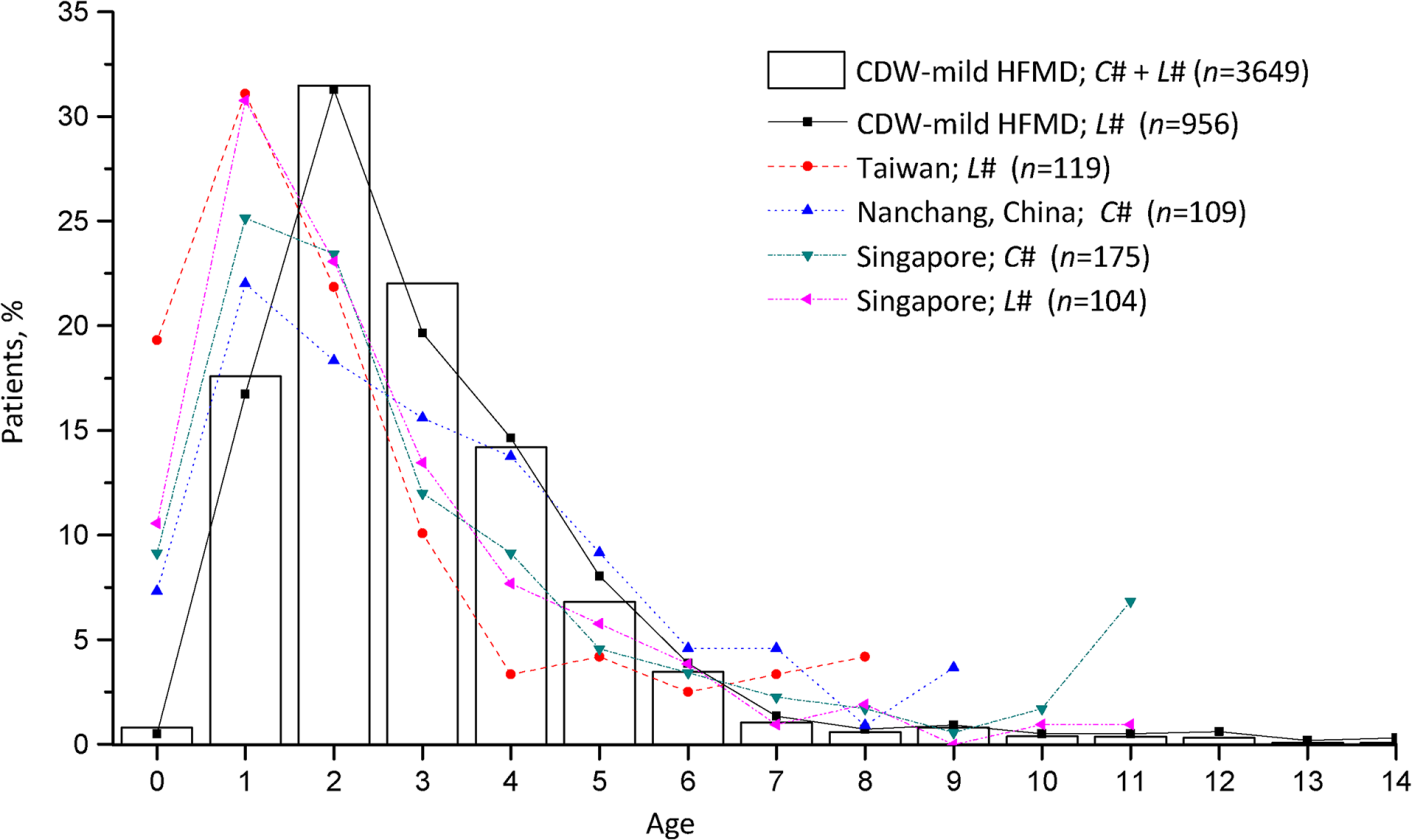
Age distribution of mild HFMD from the Clinical Data Warehouse System and other reports. *C*#, probable data; *L*#, laboratory confirmed data.

Males (62.4%) were slightly more affected than females (37.6%), and the male to female ratio was 1.6: 1. Of the 3649 patients, almost all (92.9%) were children under 5 years of age, with 71.9% falling into the age group 0–3 years (Fig 2). The age of the cases ranged from 0 to 14 years with a mean of 2.9 years and a median of 1.8 years. The results showed that the peak month-specific incidence occurred in June and July (S1 Fig).

The median time from symptom onset to diagnosis was 1 day (interquartile rang, 0 to 1 day). The causative agent was identified in 956 out of 3649 outbreaks, with CV-A16 accounting for nearly half of the results and EV71 being the second most common pathogen identified. Other causative agents included various untyped enteroviruses (Table 1).

**Table 1:** Characteristics of probable and laboratory-confirmed cases of mild HFMD.

Characteristic	*n*/total (%)
**Enterovirus serotype(N = 956)**	
**CV-A16**	405/956 (42.34)
**EV71**	370/956 (38.70)
**EV71 and CV-A16**	63/956 (6.59)
**Other enterovirus**	175/956 (18.31)
**Gender**	
**Male**	2277/3649 (62.40)
**Female**	1372/3649 (37.60)
**Symptoms and Signs**	
**Guide Group** ^a^	
**Extremity rash** ^b^	3475/3649 (95.23)
**Oral mucosa lesions**	3374/3647 (92.51)
**Blister**	2212/3645 (60.69)
**Fever**	2124/3644 (58.29)
**Loss of appetite**	1879/3644 (51.56)
**Rhinorrhea**	905/3644 (24.84)
**Cough**	501/3642 (13.76)
**Potential Signs Group** ^c^	
**Sialorrhea**	284/3642 (7.80)
**Constipation**	281/3642 (7.72)
**Lethargy**	221/3644 (6.06)
**Dysphoria**	163/3644 (4.47)
**Vomiting**	146/3645 (4.01)
**Sputum**	86/3644 (2.36)
**Diarrhea**	42/3645 (1.15)
**Age, yr, median(IQR)**	3 (2–4)
**Age group, yr**	
**0–5**	3390/3649 (92.90)
**6–10**	228/3649 (6.25)
**10–14**	31/3649 (0.85)
**The median time from symptom onset to diagnosis, days (IQR); N = 3641**	1 (0–1)

^a^ Guideline-mentioned symptoms (China, 2010ed.).

^b^ The extremity rashes varied among the case patients and were papular, vesicular, or both.

^c^ Potential warning signs in our previous study.

EV71, enterovirus 71; CV-A16, Coxsackie virus A16.

IQR, interquartile range.

### Clinical features and laboratory diagnosis

Distribution features of skin rashes are shown in S2 Table. The rashes were found mainly on hands, feet and the buttocks. In a few cases, chest, back and limbs were involved. In the 3649 cases, the main presenting signs of the disease were extremity rash, followed by oral mucosa lesions, blisters (with clear liquid or pus inside the blisters) and fever. Gastrointestinal symptoms occurred in approximately two-third of the cases, but neurological symptoms were infrequent, including lethargy and dysphoria. In the potential signs group, sialorrhea and constipation were the most frequent symptoms (Table 1). In addition, the oral mucosa lesions were found in almost all cases, with the typical manifestation involving petechial maculopapules, followed by vesicles (S2 Table).

### Symptoms and signs associations

**Fig 3:**
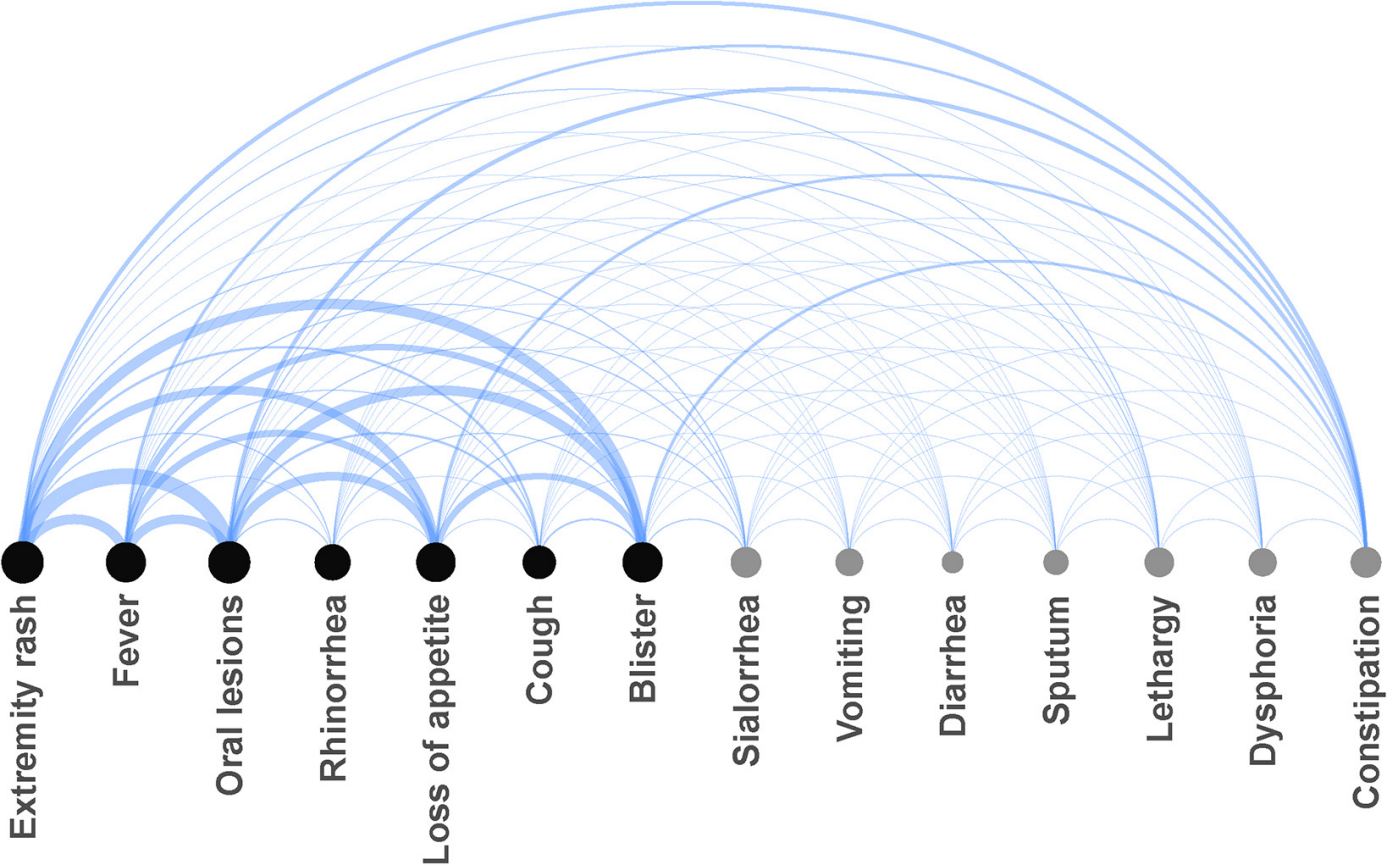
The arc diagram of symptoms and signs of patients with mild HFMD. The symptoms and signs were included in the guideline issued by China (black nodes, *Guide Group*) or previous study (grey nodes, *Potential Warnings Group*).

The arc diagram is based on a network representation of co-occurrence of clinical features in the patients with HFMD. The node colors indicate cluster memberships (Fig 3). The most frequent features in different systems were extremity rash, oral mucosa lesions, loss of appetite, cough, and lethargy. Rash with oral lesions were the most common paired signs (87.9%), followed by rash with blisters or fever (58.9%, and 54.6%, respectively). The presence of rashes and oral lesions might have contributed to the manifestation of loss of appetite. Loss of appetite with rash were the main presenting symptoms (49.0%), followed by loss of appetite with oral lesions or fever (48.7%, and 38.2%, respectively).

**Fig 4:**
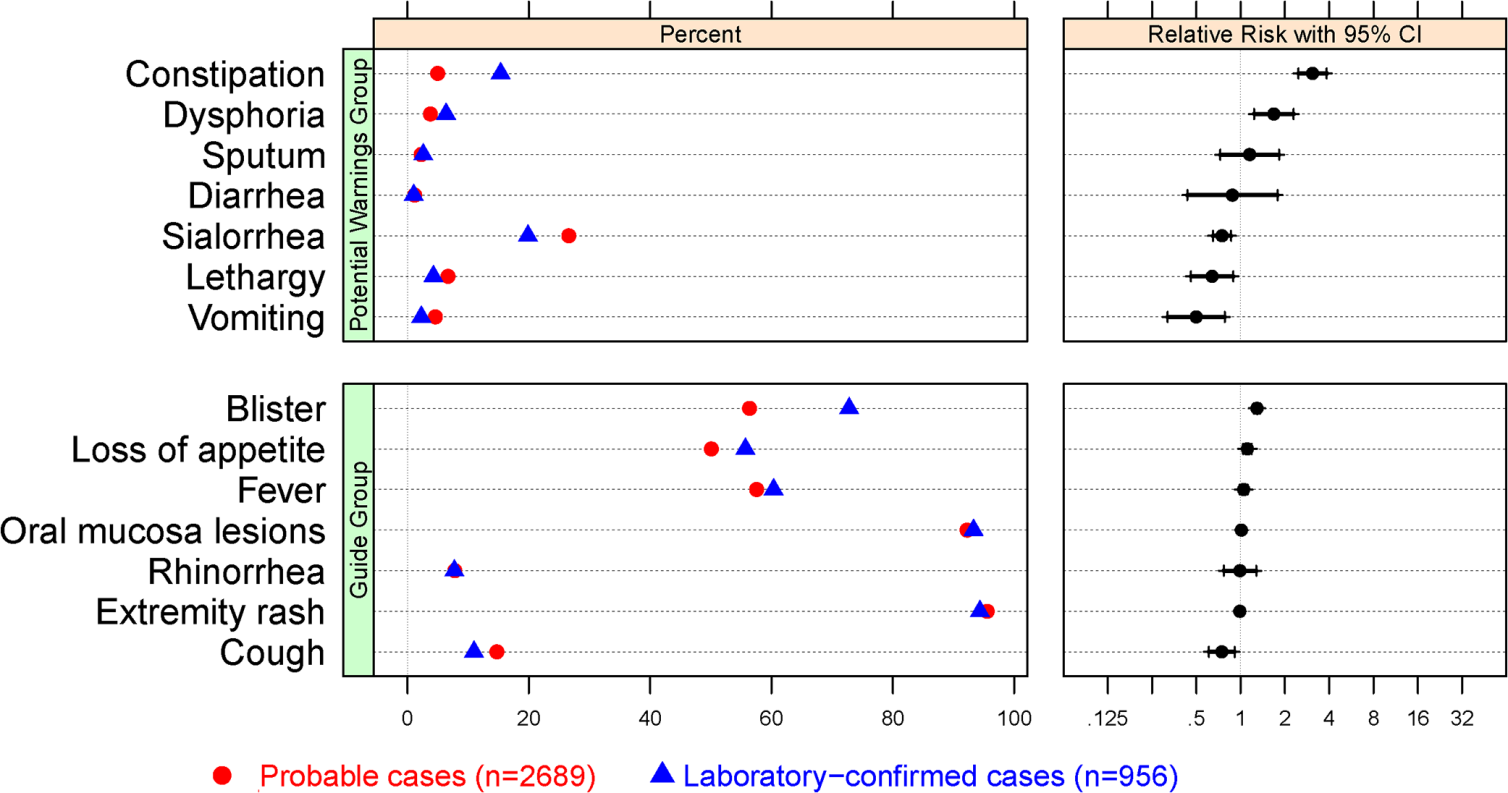
Most Frequent On Symptoms and Signs Sorted by Relative Risk. The first panel displays the percent of symptoms and signs, with different symbols for each group. The second panel displays the relative risk of a symptom on the laboratory-confirmed cases relative to the probable cases, with 95% confidence intervals for a 2 × 2 table.

The most frequent symptoms are shown in probable and confirmed cases (Fig 4). Univariable analysis showed that compared to the probable cases, the lab-confirmed cases had a higher frequency of blisters, loss of appetite, and constipation (16.43%; 95%CI (13.04%-19.81%), 5.61%; 95%CI (1.9%-9.29%), 10.39%; 95%CI (7.96%-12.82%), respectively). Logistic regression analysis also revealed that the clinical features constipation (*P*<0.0001; adjusted OR, 95%CI (2.99, 2.28–3.91)), and blister (*P*<0.0001; adjusted OR, 95%CI (2.16, 1.82–2.56)) were positively correlated with the confirmed cases. In addition, constipation had the positive predictive value (PPV) and negative predictive value (NPV) of 52% (147/281) and 76% (2552/3361), respectively in predicting lab-confirmed cases. For those presented on the blisters, the PPV and NPV for lab-confirmed HFMD were 31% (696/2212) and 18% (260/1433), respectively.

## Discussion

We evaluated the frequencies of symptoms in the largest clinical data warehouse system of patients with mild HFMD who had all guideline-mentioned symptoms (China, 2010ed). Our results show that constipation, which was overlooked in the guideline, was positively correlated with the confirmed cases. The findings suggest that constipation should be considered as potential warning signs while children are diagnosed with mild HFMD by clinical features.

### Comparison with other studies

Comparison with other studies, the age distribution of cases is similar to what has been observed elsewhere [24,25], with young children (<5 years) being mostly affected by the disease. In Fig 2, previous studies showed that the peak age-specific incidence occurred at 1 year of age [15,25,26]. Our study consistently showed that the peak incidence was identified in children 2 years of age. However, the presence of these peaks does not demonstrate a corresponding shift in the age distribution because the relatively few early papers were labeled specific data based on age distribution. Indeed, only three papers meet that requirement when searching Medline using the terms HFMD and epidemiology. The mean number cited by these articles was over 160, and corresponded to the journals with higher impact factors in the scientific fields. This study also found that the male-to-female ratio was 1.6:1. The reason for this finding is unclear but may suggest a susceptibility at the host genetic level [27].

Previous studies showed similar frequencies of rash and fever but had widely different frequencies of oral mucosa lesions, constipation, vomiting, diarrhea, loss of appetite, and so on [24,28]. For example, compared with the southern Vietnam study, the symptom vomiting was more frequent on the CDWs study (36.4% vs. 4.0%, respectively) [29]. Differences in the clinical diagnosis of HFMD may have influenced the patient populations and results. In the absence of available standardized World Health Organization case definitions and guidelines for the clinical management of cases, several countries have developed their own official guidelines [28]. In the southern Vietnam study, HFMD was defined as a febrile illness (>37.5°C) accompanied by a papulovesicular rash with a characteristic distribution (oral mucosa, extremities of limbs, buttocks) [29]. However, in China, HFMD was defined as fever with skin papular/vesicular rash on the hands, feet, mouth or buttocks; a few cases may not exhibit fever [14]. In addition, our study described the clinical features of HFMD patients without complications (i.e., mild HFMD). These factors may also affect the differences of results.

On the CDWs, confirmed cases did not have a significant association with extremity rash, oral mucosa lesions, or fever (*P* = 0.1372; *P* = 0.2793; *P* = 0.1311, respectively). The results have also been confirmed by logistic regression analysis. This is not surprising as extremity rash, oral mucosa lesions and fever were part of the definition for mild HFMD cases [14]. In our study, the logistic regression analyses demonstrated correlations between clinical features and confirmed cases. However, the presence of these clinical features in a patient does not substitute for laboratory diagnosis of HFMD. According to the clinical diagnostic criteria in China (2010ed.) [14], these correlations raise awareness that these features should be considered as potential warning signs.

The logistic regression analysis reveals that blister and constipation are important features of laboratory-confirmed HFMD. These features may be correlated with HFMD because they may occur as a result of enterovirus infections [30]. However, blisters and/or constipation are not specific to HFMD because they must appear in combination with rash, fever, and so on [14]. The blisters had a characteristic distribution with involvement of oral mucosa, hands, feet, and buttocks. In prior studies, the blisters were one of the typical clinical signs of atypical HFMD caused by CV-A6 virus [31,32]. Constipation has been reported previously as a clinical feature of HFMD. In the gastrointestinal manifestations of HFMD, previous studies paid more attention to diarrhea, which occurred infrequently (3%-10%) [1,33,34,35]. However, in our study confirmed cases presented with constipation (147/956 cases, 15.38%).

### Strengths and limitations of study

In our study, several potential limitations should not be neglected. As a descriptive study, there were relatively few items on the CDWs, although the symptoms had been determined by previous studies. Furthermore, these items were derived from the surveys in China [22], and we did not know whether our risk estimates would apply to other countries.

Another limitation of this study was that the probable cases included patients without laboratory testing and were defined by the clinical diagnosis alone. Therefore, they probably involved children with other viral infections in addition to those with HFMD infection. The precise proportion of clinical manifestations that tested positive for HFMD varied considerably between locations in the pandemic, although during the pandemic the prevalence of HFMD mirrored the prevalence of its clinical diagnosis. At any given place included in our research the varied prevalence of HFMD among children with clinical diagnosis would have also occurred temporally. However, the comparison of patients with confirmed cases to those with probable cases may provide potential warning signs while front-line clinicians manage surges of children diagnosed with mild HFMD during a pandemic.

However, our findings results must be interpreted with caution. In this study, constipation occurred exclusively in 281 (7.72%) cases. When presence of a symptom or sign occurs infrequently, the parameter estimates are often unstable in multivariable model building. In contrast, some clinical features (such as vomiting, lethargy) remain potential (and plausible) warning signs of mild HFMD and should also not be neglected in clinical diagnosis. Finally, we presented results from a retrospective study, and some bias might exist; for example, we could not exclude the possibility of residual confounding such as weather and personal biases of symptoms in real-world clinical settings [36,37], despite our multivariable models had adjusted for some covariates such as age, sex, and symptoms onset data (month).

Our study has several additional strengths. To our knowledge, this is the largest case series study of symptoms of mild HFMD. Our study had all the guideline-mentioned symptoms. This study included 21 provinces, which were geographically widely distributed throughout China. On the CDWs, each case was reviewed at least twice to ensure accurate data entry as well as correct and complete diagnoses. In addition, the items of symptoms were confirmed based on review of typical cases and discussion by clinical experts.

## Conclusions

The most frequent symptoms are reported in the clinical data warehouse, from 3649 patients with mild HFMD, who had complete guideline-mentioned information. Knowledge of the symptoms involved may provide potential warning signs while front-line clinicians manage surges of children diagnosed with mild HFMD during a pandemic.

## Supporting Information

S1 Fig Monthly distribution of mild HFMD in the Clinical Data Warehouse System. (TIF)

S1 Table Geographical distribution of laboratory-confirmed cases of mild HFMD by Chinese province. (DOC)

S2 Table Distribution features of extremity rash and oral mucosa lesions in 3649 patients. (DOC)
