# Supplementary figures and images for: Clinical Features for Mild Hand, Foot and Mouth Disease in China
Source: PLoS One. 2015 Aug 24;10(8):e0135503. doi: 10.1371/journal.pone.0135503 (PMC4547800; doi:10.1371/journal.pone.0135503)

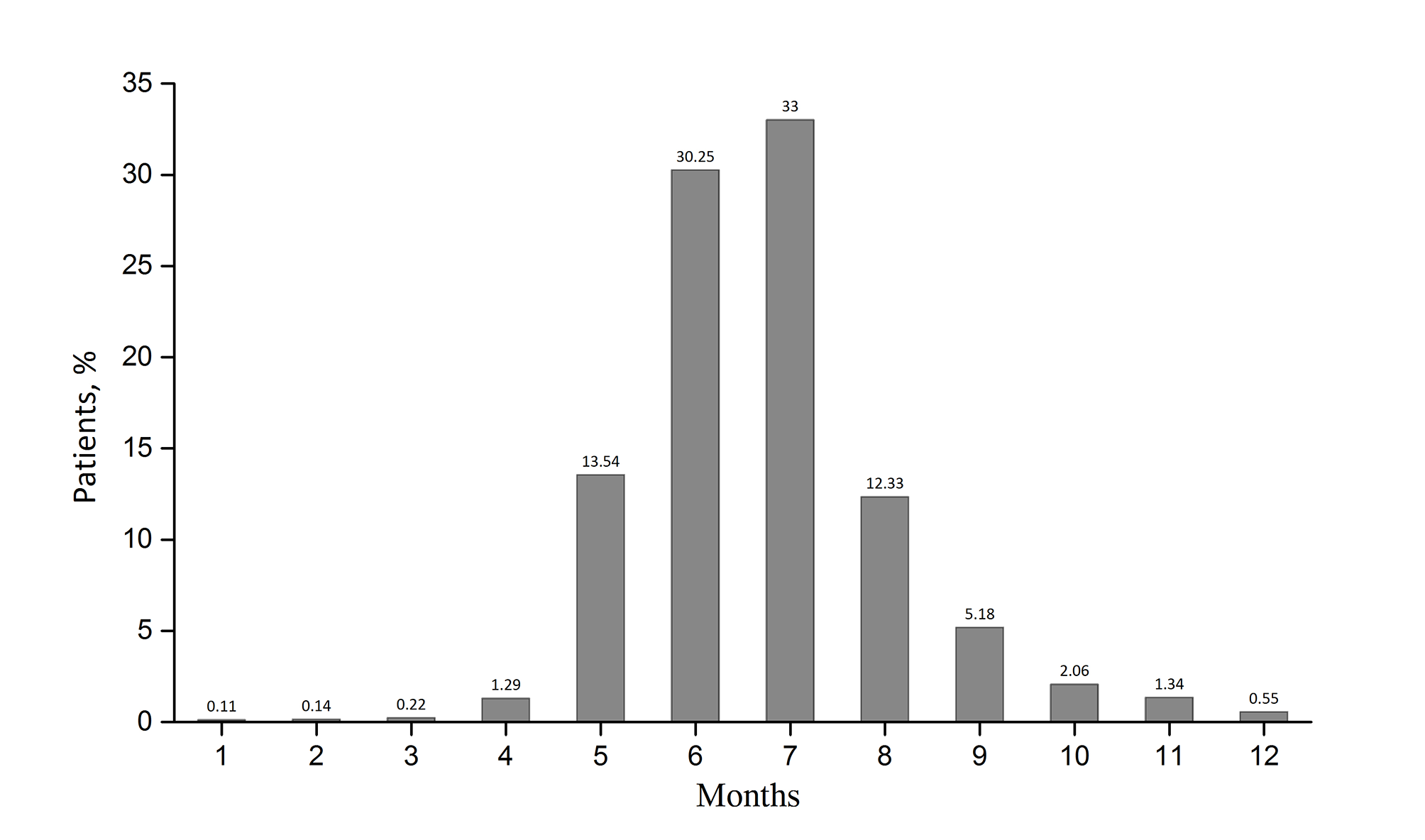

Supplement: S1 Fig — (TIF) [file pone.0135503.s001.tif]
