# Supplementary material for: Clinical Features for Mild Hand, Foot and Mouth Disease in China
Source: PLoS One. 2015 Aug 24;10(8):e0135503. doi: 10.1371/journal.pone.0135503 (PMC4547800; doi:10.1371/journal.pone.0135503)
Supplement: S1 Table — (DOC) [file pone.0135503.s002.doc]

**S1 Table.** Geographical distribution of laboratory-confirmed cases of mild hand, foot, and mouth disease by Chinese province

| **Location** | **Total** | **% Laboratory-confirmed cases** |
| --- | --- | --- |
| Hebei | 556 | 41.9 |
| Fujian | 502 | 25.5 |
| Beijing | 379 | 29.0 |
| Guizhou | 244 | 38.5 |
| Inner Mongolia Autonomous Region | 200 | 2.0 |
| Jilin | 194 | 41.2 |
| Shenzhen | 182 | 10.4 |
| Chongqing city | 181 | 7.2 |
| Hubei | 173 | 39.9 |
| Xinjiang Uygur Autonomous Region | 167 | 13.8 |
| Gansu | 148 | 6.1 |
| Guangxi Zhuang Autonomous Region | 128 | 0 |
| Jiangxi | 109 | 26.6 |
| Sichuan | 96 | 66.7 |
| Henan | 80 | 3.8 |
| Tianjin | 72 | 88.9 |
| Shanghai | 66 | 0 |
| Anhui | 63 | 7.9 |
| Hunan | 61 | 6.6 |
| Shaanxi | 39 | 10.3 |
| Yunnan | 9 | 11.1 |
| **Total** | **3649** | **26.2%** |
