# Supplementary material for: Clinical Features for Mild Hand, Foot and Mouth Disease in China
Source: PLoS One. 2015 Aug 24;10(8):e0135503. doi: 10.1371/journal.pone.0135503 (PMC4547800; doi:10.1371/journal.pone.0135503)
Supplement: S2 Table — (DOC) [file pone.0135503.s003.doc]

**S2 Table**. Distribution features of extremity rash and oral mucosa lesions in 3649 patients.

| **Sign** | **No.** | **%** |
| --- | --- | --- |
| Extremity rashes^a^  *Site* |  |  |
| Hands | 3319 | 91.0 |
| Feet | 3168 | 86.8 |
| Buttocks | 1921 | 52.6 |
| Anal area | 857 | 23.5 |
| Chest, Back and Limbs | 447 | 12.2 |
| Oral mucosa lesions^b^  *Type^c^* |  |  |
| Petechial maculopapules | 2682 | 73.6 |
| Vesicle | 448 | 12.3 |
| Ulcer or Erosion | 243 | 6.7 |

^a^ The skin rashes varied among the case patients and were papular, vesicular, or both.

^b^ 5 cases were excluded because available information was missing.

^c^ The most typical manifestation of oral mucosa lesions was recorded
